# Responding to foot health needs of people experiencing homelessness: the role of a publicly funded community-based podiatry service

**DOI:** 10.1186/s13047-022-00518-7

**Published:** 2022-02-16

**Authors:** Robyn M. Mullins, Rebecca E. Mannix, Nicole J. Marshall, Virginia J. Lewis

**Affiliations:** 1 Centre for Health Systems Development, Australian Institute for Primary Care and Ageing, La Trobe University, Plenty Rd & Kingsbury Dr, Bundoora, VIC 3086 Australia; 2 cohealth Melbourne, 53 Victoria St, Melbourne, VIC 3055 Australia; 3 cohealth Kensington, 12 Gower St, Kensington, VIC 3031 Australia

**Keywords:** Homelessness, Foot complaints, Podiatry, Rough sleepers, Community health

## Abstract

**Background:**

People experiencing homelessness are known to suffer from poor health and can be reluctant to seek healthcare except in crisis. Foot and ankle problems are a concern; as well as causing discomfort and pain, they may escalate from a minor problem to a very serious one without timely and appropriate treatment. Little is known about the foot and ankle problems of people experiencing homelessness.

This paper describes a podiatric service specifically for people experiencing homelessness, which includes a fixed site as well as outreach services. The service operates as part of the Homelessness Team program at Cohealth, a large community health service in Melbourne.

**Methods:**

The study used routinely collected data. Every person who was seen by the podiatrist in the Cohealth Homelessness Team in 2019, whether on site or on outreach, was included in the study (*n* = 295). Of these, 156 were attending for the first time and 139 were returning clients. People who used the service were predominantly rough sleeping (45.2%), with 32.2% in unstable or insecure housing and 22.6% recently housed.

**Results:**

Skin and nail pathologies (68.1%), inadequate footwear (51.9%) and biomechanical issues (44.1%) were the most common presentations. People sleeping rough were particularly likely to present with biomechanical issues (50.8%), acute wound care needs (17.4%) or traumatic injury (10.6%). Most people presented with more than one issue (mean = 2.4), and new clients (mean = 2.53) and those rough sleeping (mean = 2.69) had more issues than others.

Outreach was the most effective way to reach clients in the most difficult circumstances (48.9% of those in unstable housing, 34.8% of rough sleepers). Most of the clients (81.4%) had connections with other services offered by Cohealth, such as social work or physiotherapy.

**Conclusions:**

This study demonstrated that reaching and intervening on foot and ankle problems of people experiencing homelessness who may not seek care on their own could be achieved through a publicly funded health service, using simplified pathways to care including outreach. In addition to the long- and short- term benefits of the immediate podiatric treatment, building trust and connections through footcare may provide an entry point into accepting other health and welfare services.

## Background

### Homelessness and health

It is well established that experiencing homelessness is associated with poor physical health, even in affluent countries with universal health care such as Australia [1]. People experiencing homelessness are more likely than others to suffer from physical issues, including infectious diseases, musculoskeletal disorders, chronic disease and skin and foot problems, as well as having issues with mental health and problems with drugs and alcohol [2]. Poor health outcomes can be exacerbated by seeking help late, after a crisis occurs. Davies and Wood identify three types of barriers to health care: personal barriers such as giving priority to immediate needs such as food and shelter, practical barriers such as getting to appointments, and relationship barriers such as being judged by health professionals [2]. In this paper we look at a podiatry service which aims to overcome barriers to improve the foot and ankle health of people experiencing homelessness in Melbourne, Australia.

### Homelessness in Australia

There is no consensus on a definition of what constitutes “homelessness”, which is dependent on cultural and historical factors. In Australia, it is acknowledged that homelessness goes beyond not having a roof over one’s head, or “houselessness”, to not having a “home”. In 2012, the Australian Bureau of Statistics (ABS) adopted a statistical definition of six categories of homelessness ranging from “improvised dwellings, tents or sleeping out” (sleeping rough) through to severely overcrowded dwellings [3]. Based on this definition, 2016 Census data identified nearly 25,000 people experiencing homelessness, including 1123 who were sleeping rough [4].

In this paper, we have not utilised this statistical definition and measurement of homelessness but have adapted the broader cultural definitions first proposed by Chamberlin and MacKenzie [5] which classifies homelessness as primary (no conventional accommodation), secondary (unstable) and tertiary (insecure) accommodation (described in the Method section).

### Foot and ankle health and homelessness

Foot and ankle health is an important component of overall health, and podiatric issues range from acute issues to chronic conditions. Foot and ankle problems are of concern not only because of the discomfort they can cause, but also because of the potential for an issue to escalate from a minor problem to a serious one if appropriate treatment is not accessed early enough. As one example, a simple abrasion which is not cared for appropriately may develop into cellulitis, which accounts for an estimated 11% of preventable hospitalisations in Australia [6].

Expected common presentations for podiatric care include skin and nail pathologies, foot infection, foot pain, ulceration, and diabetes-related foot concerns; however, there is little data available on the overall prevalence of foot conditions in the general population, or about conditions seen by podiatrists. Given this, it is not surprising that there is also little information about podiatric conditions in specific disadvantaged populations, such as people experiencing homelessness. A systematic review of the foot and ankle problems of people experiencing homelessness identified 17 studies, although these dated back to 1979 and some were described as methodologically weak [7]. This review found estimates of the prevalence of specific conditions among homeless populations varied greatly in different studies, which is not surprising given the differences in study methods and design. In some instances, study participants had presented specifically for foot care, but other studies involved screening in settings such as shelters. Additionally, some methods were based on self-report, while others included a clinical examination. Overall, the reviewed studies pointed to high levels of corns and calluses, nail pathologies and infections in homeless populations, as well as higher levels of tinea pedis, foot pain and difficulties walking than in comparison groups that were housed.

A number of studies have considered causal risk factors for poor foot and ankle health. Correctly fitted footwear is important to prevent friction, trauma, and musculoskeletal injury, but it has been demonstrated that many adults in the general population (63–72%) do not wear correctly fitted shoes [8]. The potential impacts of this are exacerbated amongst people experiencing homelessness who lack the resources to access appropriate footwear even if they would like it. For example, people using a specific homeless health service in the United States (the Bowery, New York city) were more likely than clients at a private clinic to be wearing shoes up to 1.5 sizes wrong for their feet [9]. The benefits of correctly fitted footwear have been documented. A small study has shown that people experiencing homelessness reported a reduction in pain as well as improved walking speeds six weeks after being provided with correctly fitted sports shoes [10]. Well-fitted footwear is particularly important for anyone with diabetes as they may be unaware of ulceration if it occurs due to peripheral neuropathy [11]. The International Working Group on the Diabetic Foot recognises the importance of preventing foot ulcers, given their very high prevalence and risk of recurring, and the negative impact on individuals and health systems [12]. Foot ulceration has been reported as a major risk factor for hospital admission and amputation in individuals with diabetes [13].

### Use of podiatry

In Australia, which has a population of approximately 25 million, there were 3.3 million publicly funded podiatric visits [14] and 2.8 million privately funded ones [15] in the financial year 2017–2018. This points to a high level of demand for podiatric care across the community. However, many people report that podiatry is too expensive and that they would go to a general practitioner if they had a problem with their feet [16]. Foot pain in itself is not enough to motivate most people to attend a podiatrist, and men and young people may be least likely to act on their pain [8, 17]. Even among a group with a chronic health condition known to cause foot problems, rheumatoid arthritis, the decision to see a podiatrist was not seen as a priority [18]. These findings suggest that those who are marginalised for a variety of reasons including precarious housing status, may be reluctant to attend mainstream health services for foot care.

### A podiatry service for people experiencing homelessness

This study reports on a no cost podiatric service offered by a large community health service to people experiencing any type of homelessness, whether they be sleeping rough, in crisis accommodation, couch surfing or other inadequate accommodation. Care is also provided to those who have been recently housed, but who are still considered to be at risk of experiencing homelessness again unless appropriate supports are in place.

Cohealth is one of Australia’s largest community health services, operating at multiple sites across 10 local government areas in Melbourne. It offers a wide range of low-cost health and welfare services including general practice, nursing, dental, physiotherapy, social work, occupational therapy, dietetics, diabetes nurse educators and counselling [19]. Podiatry services are offered at seven sites as part of an integrated, multi-disciplinary primary health care model. The types of services offered at each site are tailored to the local community needs. The Homeless Team operates from a site in central Melbourne and specifically targets people experiencing issues with accessing and maintaining adequate housing. As well as fixed site services, the Homelessness Team also operates an assertive outreach model, offering a drop-in health service at places where people experiencing homelessness gather. One podiatrist works as a sole practitioner within a multidisciplinary team and can refer clients to services at any Cohealth site. People can self-refer to the homelessness podiatry service or be referred there by Cohealth practitioners working in other areas, other homelessness organisations, or other health services.

The Cohealth homelessness podiatry service operates four days per week, partly in a central city location and partly through outreach. People experiencing any type of homelessness as well as those who are recently housed but still considered to be at risk of homelessness--due to recent experiences of homelessness, or the presence of complex mental health and/or substance abuse issues--are able to make an appointment to see the podiatrist. Care is taken to ensure that appointments are well-spaced out to allow time for some of those who are sleeping rough to be seen opportunistically, rather than being expected to make and attend an appointment. As well as allowing for immediate treatment, this can help build rapport and assist in improving health literacy. No appointment is required when the podiatrist conducts outreach at other services.

Cohealth works collaboratively with other not-for-profit organisations providing health care and support to people experiencing homelessness. Bolton Clarke (formerly the Royal District Nursing Service) is one of Australia’s largest not-for-profit providers of community nursing services, and operates a Homeless Person’s Program (HPP), working collaboratively with a range of providers [20, 21]. Accessing adequate quality footwear can be challenging for Cohealth’s clients. In order to provide good quality sports shoes (mainly second-hand) and new socks to clients in need, Cohealth partners with Footscape, a local charity founded by a podiatrist to address footwear inequality [22].

### Aim

The purpose of this paper is to describe the types of clients who received help from the homelessness podiatrist, and the conditions for which they sought help. Any link between the type of homelessness they were experiencing at the time of presentation and the presenting conditions was explored. Additionally, given existing reports of barriers to healthcare seeking among people experiencing homelessness, the way people first came to access the service is explored.

## Method

### Setting

The study was conducted at the podiatry clinic in an inner-city community health service specifically for people experiencing homelessness. Data was also collected when the podiatrist conducted outreach at other homelessness services.

### Participants

Routinely collected data from everyone who was seen by the podiatrist in the Cohealth Homelessness Team in 2019, either on-site or on outreach, was included in this study. Data were taken from the first visit by new clients, and from the first visit in 2019 for returning clients. Thus, each client is only represented in the data once.

### Design and data collection

Information is collected from all attendees at the community health service as part of routine data collection to allow for service evaluation and research. All clients are asked at the time of registration to consent to their deidentified data being used for service provision, and for quality improvement and research. The project had ethical approval from both La Trobe University (HEC21028) and Cohealth (HEAG2101).

All data collected at the podiatry clinic in 2019 were analysed to develop a profile of the clients using the service, look at the types of problems clients presented with, how they found out about the podiatry service and whether they had connections with other parts of the community health service. The podiatrist who has provided the clinical care in the homelessness service for 10 years (REM) reflected on her experience in response to the question “What would you tell someone who wants to set up a similar podiatry service?”

Two Cohealth podiatry staff (REM and NJM who is the Practice Excellence Coach for the podiatrists across all Cohealth sites) reviewed clinical notes and coded the presenting complaints. Table 1 describes the categories used.

**Table 1:** Classification of presenting issues

Condition	Description
Skin and nail pathologies	hyperkeratosis (callous), heloma durum (corn) and all other skin and nail pathologies),
Biomechanical	all musculoskeletal lower limb presentations
Acute wound care	including blisters and all stages of acute wounds
Chronic wound care	not progressing through normal wound healing stages, and/or present for more than four weeks
Fungal	including tinea pedis of skin or nail
Infection (excluding Pitted keratolysis)	Diagnosed on clinical visual signs only
Pitted keratolysis (PK)	categorised separately from infection as it is perceived to be more common among those sleeping rough than in the rest of the population [23] and is preventable if treated appropriately
Traumatic injury	including lacerations, grazes, scratches, glass, injection site trauma and self-harm
Diabetes care	Type 1 and Type 2
Vascular conditions	including peripheral arterial disease, peripheral venous disorders
Footwear	provided when possible if the client attended lacking footwear altogether, or with footwear which was inadequate due to either the fit or condition

Categorisation was cross-checked and uncertain data were resolved through discussion between the clinically qualified authors and de-identified data were entered into an Excel spreadsheet by the Cohealth staff (NJM, REM).

The source of referral to the podiatry service was classified as:

- Specialist nurse
- Internal Cohealth staff
- External homelessness support service
- Self or a friend
- Outreach by the Cohealth podiatrist

Additionally, whether clients had connections with other programs or services in Cohealth was recorded as “yes, had connections” or “no, did not have other connections”.

University staff (RMM) assigned codes to housing status and to source of referral. Housing status was classified based on the cultural definition of homelessness first proposed by Chamberlain and MacKenzie in 1992 [5].

- Primary homelessness describes those without conventional accommodation, such as sleeping rough or squatting
- Secondary homelessness is unstable housing, such as emergency accommodation and couch surfing
- Tertiary homelessness describes below standard housing, without security of tenure, such as boarding houses

People who have been recently housed (less than 6 months) are also eligible to use this podiatry service, so this was added as a fourth housing status category (Recently housed).

The categories of secondary and tertiary housing each include a range of types of accommodation; for example, a person who is experiencing secondary homelessness may be temporarily staying with relatives or could be accommodated in a boarding house with shared facilities. Thus experiencing either secondary or tertiary homelessness is unlikely to affect foot health in a consistent way. Additionally, the number of people in the secondary and tertiary categories was relatively small, so there were too few to analyse reliably. Consequently, these two categories have been combined into one category for most analyses and the housing groups described here are: rough sleeping, insecure/unstable housing, recently housed.

### Data analysis

Variables available for analysis were sex, age (< 30, 31–50, 50+), date of presentation, presenting issue, new or returning client, how the client found out about the service, whether the client was referred on to any other Cohealth service. Data were then analysed by the university staff (RMM, VJL) using SPSS 27 [24] Basic demographics including frequencies and means were calculated. Comparisons of distributions of nominal variables (demographics, presenting issues) across different groups (housing situation, new or returning client) were undertaken using chi-square. Welch’s one-way analysis of variance (for variables with unequal variances) was used to compare the mean number of issues (continuous variable) for three independent categories (housing situation), with Games-Howell post hoc comparisons. An independent t-test was used to compare the mean number of issues for new and returning clients.

Four people who did not identify as male or female (two intersex, one transgender, one did not disclose) have been excluded from analyses of effects of sex but are included in all other analyses. Their exclusion from these analyses by sex is required for the statistical analyses, and to ensure that respondents are not identifiable. Three people’s housing situation did not fit any classifications, so they are also excluded from the analyses of patterns of housing data but included in all others.

## Results

In 2019, 295 individuals received care from the homelessness podiatrist, and there were 765 visits in total. Of the clients, 156 were attending for the first time and 139 were returning clients.

### Profile of service users

The service users were predominantly male (79%), and very few clients were under the age of 30 (8.1%, mean age = 47.8). There was no significant association between the age and sex of clients (χ^2^ = 1.39, df = 2, *p* = 0.5).

As the data in Table 2 demonstrate, nearly half the clients (45.2%) were sleeping rough and almost one-quarter (22.6%) were recently housed. The others were in unstable (16.1%) or insecure housing (16.1%).

**Table 2:** Demographics by housing status

		Rough Sleeping	Unstable	Insecure	Recently Housed	χ^2^	*P*=
	***N*=**	**132**	**47**	**47**	**66**		
Total		45.2%	16.1%	16.1%	22.6%		
30 or under	24	41.7%	37.5%	16.7%	4.2%	38.45	.001
31–49	141	53.2%	14.9%	19.9%	12.1%		
50+	127	37.0%	13.4%	11.8%	37.8%		
Male	228	48.7%	16.7%	16.2%	18.4%	13.61	.003
Female	60	30.0%	13.3%	16.7%	40.0%		
New client	153	48.4%	20.9%	15.0%	15.7%	12.76	.006
Return client	139	41.7%	10.8%	17.3%	30.2%		

Age, sex, and new/repeat visit were all associated with the type of homelessness clients were experiencing. Men, new clients, and those aged 30–49 years were most likely to be sleeping rough.

### Presenting issues

The issues clients presented with at their first visit in 2019 were coded into 11 categories, as described above.

As the data in Table 3 indicate, more than two-thirds of clients presented with skin and nail pathologies, and more than half attended without adequate footwear, having footwear that fit poorly or was in poor condition, or lacking footwear altogether. Biomechanical issues and fungal infections were also quite prevalent, with nearly half presenting with biomechanical issues and nearly one-quarter with fungal issues. There were some significant associations between particular presenting issues and whether the client was new or returning. New clients presented with more acute wounds, vascular issues, and traumatic injury. Returning clients presented with more skin and nail pathologies, and diabetes. There was a tendency for more new clients to need adequate footwear, but the difference was not significant.

**Table 3:** Presenting conditions for new and returning clients (percentages)

	Number with condition	Total % *N* = 295	New client *N* = 156	Return client *N* = 139	χ^2^=	*P*=
Skin and nail pathologies	201	68.1	62.8	74.1	4.31	.038
Footwear	153	51.9	57.1	46.0	3.57	.059
Biomechanical	130	44.1	48.1	39.6	2.16	.142
Fungal	69	23.4	27.6	18.7	3.22	.073
Diabetes	45	15.3	8.3	23.0	12.27	.001*
Acute wound care	34	11.5	16.0	6.5	6.56	.01*
Vascular	25	8.5	11.5	5.0	4.01	.045*
Traumatic injury	17	5.8	8.3	2.9	4.03	.045*
Chronic wound care	14	4.7	2.6	7.2	3.49	.062
Infection	13	4.4	6.4	2.2	3.16	.076
Bacterial infection (PK)	7	2.4	3.8	0.7		Too small to test

*Significant at *p* < 0.05; Bonferroni correction applied to z-tests for independent proportions

There were no significant differences in the conditions with which men and women presented. A high proportion of those seen by the podiatrist on outreach had fungal problems (34.9%, χ^2^ = 10.95, df = 4, *p* = .027), however there were no other differences for those who came to the service in different ways.

The need for care for skin and nail pathologies increased with age (χ^2^ = 7.87, df = 4, *P* = .02), with nearly three-quarters of the oldest group (74.2%) requiring help in this area. The youngest clients were more likely to present with traumatic injury (20.8%) than either of the older groups (χ^2^ = 11.05, df = 4, *p* < .004), and the need for chronic wound care was very low in those aged 30–49 years (χ^2^ = 6.9, df = 4, *p* = .032). There were few clients who presented for traumatic injury (17 clients) or chronic wound care (14 clients) so although these differences are significant, they may not be very meaningful.

The presenting conditions of people in different housing situations were also considered (Table 4).

**Table 4:** Presenting conditions for clients by housing status (percentages)

	N	Total % *N* = 292	Rough Sleeping *N* = 132	Unstable/ insecure *N* = 94	Recently housed *N* = 66	χ^2^	*P*=
Skin and nail pathologies*	199	68.2	68.9^a,b^	56.4^b^	83.3^a^	13.0	.001*
Footwear	151	51.7	51.5	58.5	42.4	4.02	.134
Biomechanical	129	44.2	50.8^a^	43.6^a,b^	31.8^b^	6.42	.04*
Fungal	69	23.6	25.8	23.4	19.7	.89	.64
Diabetes	44	15.1	15.9	13.8	15.2	.186	.91
Acute wound care	33	11.3	17.4^a^	6.4^b^	6.1^a,b^	9.01	.01*
Vascular	25	8.6	10.6	6.4	7.6	1.36	.51
Traumatic injury	17	5.8	10.6^a^	3.2^a,b^	0^b^	10.78	.005*
Chronic wound care	14	4.8	5.3	3.2	6.1	–	–
Infection	13	4.5	6.8	3.2	1.5	–	–
Bacterial infection (PK)	7	2.4	5.3	0	0	–	–

-Cells too small to analyse

* Significant difference *p* < .05; Bonferroni correction applied to z-tests for independent proportions - each subscript letter denotes a subset of housing categories whose column proportions do not differ significantly from each other at the .05 level

There were four conditions which affected people in one housing situation more than those in other situations, and in three cases it was those sleeping rough who were most impacted. They were more impacted by biomechanical issues, traumatic injury and the need for acute wound care. It was also notable that the only cases of PK were among those sleeping rough. In contrast, those who were recently housed were more likely to need treatment for skin and nail pathologies, with 83.3% of this group presenting for this reason.

### Mean number of issues

The majority of those who attended the clinic presented with multiple issues, with only 19.3% having a single issue and the rest having up to six. Of those who had a single issue, mostly commonly it was skin and nail pathologies (47.4%), followed by biomechanical issues (26.3%) or inadequate footwear (15.8%).

The mean number of issues overall was 2.4, with most people having more than two issues and less than three. New clients presented with more issues than returning clients (2.53 cf. 2.26, t(292.4) = 2.16, *P* = .031), and those sleeping rough had more problems on average than those in unstable/insecure housing or those who were recently housed (2.69 cf. 2.18 or 2.14, F(2,173.76) = 9.14, *p* = <.001). The difference between those in unstable/insecure housing and those who were recently housed was not significant.

### How clients found out about the service

Although there is no need for clients to have a formal referral to the service, many are sent there either from other parts of the community health service or by other homeless services including specialist nurses. Housing is reported in four categories in Table 5, to illustrate the referral pathways in more detail.

**Table 5:** How the client found out about the service, by housing status (percentages)

	Total *N* = 292	Rough Sleeping *N* = 132	Unstable *N* = 47	Insecure *N* = 47	Recently Housed *N* = 66
Specialist nurses *n* = 47	16.1	13.6	21.3	25.5	10.6
Internal Cohealth *n* = 67	22.9	22.0	14.9	29.8	25.8
External service *n* = 49	16.8	14.4	2.1	19.1	30.3
Self/friend *n* = 46	15.8	15.2	12.8	14.9	19.7
Outreach *n* = 83	28.4	34.8	48.9	10.6	13.6

More than a quarter of clients were from the podiatrist’s outreach (28.4%), and just under a quarter were referred from other parts of Cohealth (22.9%). Self-referrals, nurses and external services each accounted for around 16%. As the data in Table 4 indicate, there were differences in the types of clients reached by different services (χ^2^ = 41.65, df = 12, *p* < .001). Outreach was particularly effective in reaching people in unstable housing and those who were sleeping rough.

### Clients’ connection with other services at Cohealth

Overall, four in five (81.4%) of the Cohealth podiatry clients had other connections with cohealth services. There was no difference in the connection to other Cohealth services for men and women, people of different age groups, or clients living in different types of housing. Clients who were new to the podiatry service in 2019 were less likely to have connections with other services in Cohealth (73.7%) than returning clients (89.9%) (χ^2^ = 11.47, df = 1, *p* < .001).

As the data in Table 6 show, the way in which clients were referred to the podiatry service made a difference to whether or not they had other connections (χ^2^ = 34.65, df = 4, *p* < .001). Around one-third of podiatry clients referred by specialist nurses and one-third of those seen on outreach did not have other Cohealth connections.

**Table 6:** Clients’ connections to other services at cohealth, by referral to podiatrist

Source of referral to podiatrist	Uses other Cohealth services	
	Yes	No
Specialist nurses *n* = 48	68.8	31.3
Internal Cohealth *n* = 67	100	0
External service *n* = 50	82.0	18.0
Self/friend *n* = 47	93.6	6.4
Outreach *n* = 83	66.3	33.7

### Reflections by the podiatrist

Attending a fixed site service can be very intimidating for some clients, especially if they have experienced trauma, and/or have had bad experiences receiving health care in the past. Many will be experiencing a lack of sleep and possibly a lack of food and drink. Creating a calm welcoming physical environment, possibly with tea and coffee available, can reduce the intensity of the experience of approaching a service.

Ensuring that there is adequate time between appointments also contributes to creating a safe space for clients. This means the interaction between podiatrist and client does not need to be rushed which contributes to a calm treatment environment for both parties. If the podiatrist is overworked and stressed this can affect people who have experienced trauma in particular, as they are often hyperaware of surroundings. More relaxed spacing of appointments also allows potential clients who present at the service to be seen promptly, rather than being expected to come back for an appointment in the future. It can also be helpful to offer special sessions for groups such as women or young people who may be uncomfortable sharing spaces with a wide range of others.

Developing relationships with other services and programs offered to people experiencing homelessness is vital as case workers and other service providers can be very reluctant to send their clients to a health professional they do not know. Building trust is a time consuming but crucial step in encouraging referrals.

Providing outreach at other services can help the service to reach a new group of clients, and the visible presence of “the foot girl” prompted action on issues which may otherwise be neglected, such as clients who are limping. Additionally, the podiatrist’s presence at a site can help identify issues which maybe specific to that site – for example, a high level of tinea pointed to the need for improvements to the showering facilities in one service.

Podiatry can provide a gentle easing into physical touch and additional care for clients who are very reluctant to accept either of these. One client is known to have attended the podiatric service 19 times before being willing to accept any other assistance. The client had pitted keratolysis and would not shower at any of the services but did consent to footcare. Treatment by the podiatrist prevented the worsening of the pitted kerotolysis but also enabled other staff at the site to develop the client’s trust. Ultimately the client reached the point where he could be housed in supported accommodation.

## Discussion

This study identified that the most common presenting conditions in an inner-city podiatry service for people experiencing homelessness were skin and nail pathologies, the need for adequate footwear, biomechanical issues, and fungal infections. There were some differences in the presenting conditions of clients living in different housing situations, providing insights into the links between living conditions and podiatric issues.

Those who were sleeping rough were particularly prone to biomechanical problems, traumatic injury, and acute wounds, and although there were very few cases of pitted keratolysis, which is sometimes regarded as a disease of the past, they all occurred in this group [25]. People sleeping rough have many issues which can affect their foot health, including being constantly on their feet, difficulty showering or maintaining foot hygiene, and are exposed to bacterial, viral, and fungal infections [23, 26]. Some of their foot issues would be simple to address with antiseptic and dressings; however, it can be difficult for clients to carry wound care equipment, and they may experience their possessions being lost, stolen or even confiscated due to strict regulations to manage belongings left on the street [27, 28].

A lack of storage facilities and the risk of having their belongings confiscated means that many people sleeping rough need to keep their possessions with them at all times [28]. Frequently carrying heavy loads without proper footwear and equipment has been shown to have negative biomechanical impacts in military and other service populations, where footwear and training are provided to ameliorate the problems [29], and we have demonstrated that biomechanical problems are prevalent among those sleeping rough. The cohealth podiatrist was able to advocate successfully for some hiking backpacks to be distributed to try to alleviate this issue in the short-term; additionally, as a community health centre [30] Cohealth works with other organisations to advocate for changes to policies and systems that affect communities’ health and wellbeing, particularly for vulnerable populations.

The findings suggested that presentations for skin and nail pathologies were particularly prevalent among those who been recently housed. This may reflect one of the underlying benefits argued for a “Housing First” model to address homelessness [31]. It is beyond the scope of this paper to describe the Housing First model, except to note that, if clients are housed, they may then be better equipped physically and emotionally to address some of the issues which had not taken priority for them previously, including their foot and ankle health. This also highlights how important it can be to provide continuity of healthcare as someone transitions through different housing, as they will still have health issues which require support [32]. Publicly funded podiatry and other health services need to be accessible to a person as they transition to secure housing, including recognising that the goal of sustaining secure appropriate housing is not always achieved the first time it is experienced [33, 34], With its integrated services and links to other key providers in the sector, Cohealth is able to provide and support wrap-around continuous healthcare that is familiar to the person as they experience housing transitions.

There are limits to the type of care that can be provided in a homelessness podiatric service, given the living conditions of clients, particularly those sleeping rough. This means that treatments and interventions that might be commonplace in private or facility-based health services may not be practical. For example, strapping a client’s feet is not appropriate in cases where it is almost impossible for the client to keep their feet clean and dry, and they often get confused about when and how to remove the strapping. Heat packs are also impossible for clients who do not have access to a microwave, as are icepacks for those without freezers. Even recommending rest can be difficult, as people need to move to access food or support services.

More than half of the people who attended this service needed assistance to access adequate footwear, including 42% of those who were recently housed. Adequate footwear is vital for people experiencing homelessness, as walking is often the primary mode of transport, with many people regularly carrying heavy loads; proper footwear can help to prevent overuse injuries, acute wounds, falls and chronic injuries [35, 36]. Shoes may wear out or be lost or stolen, but (anecdotally) some clients are reluctant to accept additional footwear if it means adding to what they need to carry. Accessing some form of housing may remove this barrier.

A key role of the Cohealth Homelessness Team approach is to offer a primary healthcare outreach service, going out to where people are located, and assertively reaching those who may otherwise never receive primary health care, including podiatry. This study found that outreach work was particularly effective at engaging those who were either sleeping rough or in the most difficult housing conditions. The presence of the podiatrist was a prompt to both clients and staff to have issues addressed which may otherwise be neglected, and additionally, the podiatrist could identify some systemic issues at specific services which could be addressed.

The importance of partnerships and networks of providers working to support the health and wellbeing of people experiencing homelessness and insecure housing is highlighted in the findings. As well as long-term primary healthcare programs delivered by Bolton Clarke HPP and Cohealth, other service connections are supported by formal and informal agreements between a range of government and non-government organisations. Some of these organisations can provide resources to enable Cohealth to respond to the needs of clients where it has no funding available. For example, Cohealth is associated with Melbourne-based charity Footscape, which has a mission to “assist disadvantaged individuals and communities predisposed to debilitating foot pathology” [22]. The material aid arm of this charity supplies some of the shoes and socks needed for distribution by Cohealth [37].

Podiatry is an essential component of primary healthcare for people experiencing homelessness and insecure housing. Problems with the feet cause discomfort or pain, and in some cases can lead to sepsis, amputation, or mortality. Given walking is the prime mode of transport for most people experiencing homelessness, podiatric problems can compound all the other difficulties they face. Additionally, many foot conditions can lead to serious problems if left untreated, which impacts negatively on the individual, as well as increasing costs to the health system. The decision to access podiatric care can be complicated for anyone; as for any health behaviour, it requires people to perceive a problem, believe it can be helped by podiatry and know how to access podiatric care [38]. People experiencing homelessness self-report fewer foot conditions than are detected clinically [39], and it has been suggested that some people are reluctant to attend a podiatrist in particular because of embarrassment about the state of their feet which may be exacerbated those who are experiencing homelessness [23]. This makes simplifying pathways to seeing a podiatrist critical. In this study fewer than one in seven people indicated that they had sought out the podiatry service themselves (or via a friend). All others attributed their attendance to either a health/welfare service recommendation or outreach by the podiatrist, further reinforcing the importance of creating a strong network collaborative service system for this population.

The podiatric service described here operates solely to address the needs of those experiencing homelessness and insecure housing. As well as a possible reluctance to seeking podiatric care, those experiencing homelessness are also known to seek help late for medical complaints. Many people experiencing homelessness have suffered previous trauma and it has been suggested that general practitioners should not conduct a physical exam at a first visit unless it is completely necessary, as it may exacerbate trauma [2]. Making podiatric care easy to access, including through assertive outreach to people sleeping rough or residing in unstable or insecure housing as well as via services and programs offered at other organisations, is essential to reach those who may not seek care on their own. Once people have accepted and experienced this care positively, it may smooth the way for them to access other health and welfare services that are available to them. If people who are experiencing homelessness can be introduced to podiatry as health care which can offer immediate interventions and positive outcomes, this may serve as a gentle introduction to physical treatment for those who are cautious about medical care. Cohealth staff, including the podiatrists, adopt a trauma-informed approach, as priority populations for services are people who experience inequity in health and access to care through their experiences of a range of social determinants of health [40]. All interactions with the podiatry service are aimed at building clients’ confidence to seek care for other needs, including introducing clients to other staff and facilitating appointments with other services.

## Strengths and limitations

The key strength of the study was that it provided insight into the foot and ankle health care needs of people who are often not recognised in health services research. The study was able to report on a large number of clients because the service is located in the central business district where many people sleeping rough live.

Due to the nature of this group we did not use a specific existing scale to classify their presenting complaints. In some instances we wanted to look in detail at the type of condition – for example, traumatic injury was coded separately from “wounds”, as it was perceived to be of particular concern for those who were rough sleeping. On the other hand, all biomechanical issues are presented as one category, as the specific nature of the issue is not of concern. This may limit the comparability of our data to other studies conducted with other populations.

The categorisation of a client’s homelessness (rough sleeping, insecure or unstable housing/newly housed) was based on what was recorded at the time of their presentation at the podiatrist. People who are homeless are known to cycle through different types of accommodation [34], so some may have acquired their problem while living in different conditions. However, where there are significant differences in the presentations by clients in different categories they fit with what would be expected of those living conditions, so this may not have impacted the results.

### Recommendations for future research

Future research could explore strategies to intervene to prevent some of the common issues identified in this paper and adapt treatments that could be implemented with people experiencing homelessness. The role of podiatry as an entry point to other healthcare for people who face the greatest barriers to access could be explored further.

## Conclusions

This study demonstrated that reaching and intervening on foot and ankle problems of people experiencing homelessness--who may not seek care on their own--could be achieved through a publicly funded health service, using simplified pathways to access care including outreach. In addition to the long- and short- term benefits of the immediate podiatric treatment, building trust and connections through footcare may provide an entry point into accepting other health and welfare services. The provision of appropriate storage facilities may also help lessen the prevalence and impact of biomechanical issues for those sleeping rough, many of whom frequently carry heavy loads.

## Data Availability

Data is not available due to its confidential nature.

## References

[1] Fazel S, Geddes JR, Kushel M (2014). The health of homeless people in high-income countries: descriptive epidemiology, health consequences, and clinical and policy recommendations. Lancet.

[2] Davies A, Wood LJ (2018). Homeless health care: meeting the challenges of providing primary care. Med J Aust.

[3] Australian Bureau of Statistics. Discussion Paper: Methodological Review of Counting the Homeless. 2006.

[4] Australian Bureau of Statistics. 2049.0 - Census of Population and Housing: Estimating homelessness, 2016. Canberra: ABS; 2018.

[5] Chamberlain C, MacKenzie D (1992). Understanding contemporary homelessness: issues of definition and meaning. Aust J Soc Issues.

[6] Australian Commission on Safety and Quality in Health Care. https://www.safetyandquality.gov.au/sites/default/files/migrated/1.3-Cellulitis.pdf Accessed 12 August 2021.

[7] To MJ, Brothers TD, Van Zoost C. Foot conditions among homeless persons: a systematic review. PLoS ONE. 2016;11(12):e0167463. 10.1371/journal.pone.0167463 PMC5147925 27936071

[8] Buldt A, Menz H. Incorrectly fitted footwear, foot pain and foot disorders: a systematic search and narrative review of the literature. J Foot Ankle Res. 2018;11(1):43. 10.1186/s13047-018-0284-z PMC6064070 30065787

[9] Schwarzkopf R, Perretta DJ, Russell TA, Sheskier SC (2011). Foot and shoe size mismatch in three different New York City populations. J Foot Ankle Surg.

[10] Moes J (2019). Proper fitting shoes: reducing pain, increasing activity, and improving foot health among adults experiencing homelessness. Public Health Nurs.

[11] Chicharro-Luna E, Ortega-Avila AB, Requena-Martínez A, Gijon-Nogueron G (2021). Fit for purpose? Footwear for patients with and without diabetic peripheral neuropathy: a cross-sectional study. Prim Care Diab.

[12] International Working Group on the Diabetic Foot. https://iwgdfguidelines.org/guidelines/guidelines/ Accessed 12 August 21.

[13] Beaney AJ, Nunney I, Gooday C, Dhatariya K (2016). Factors determining the risk of diabetes foot amputations – a retrospective analysis of a tertiary diabetes foot care service. Diabetes Res Clin Pract.

[14] Australian Institute of Health and Welfare (2020). Allied health and dental services.

[15] Australian Institute of Health and Welfare (2020). Primary health care.

[16] Australian Podiatry Association. 2020 Foot Health Survey. https://www.podiatry.org.au/documents/item/2319. .

[17] Menz HB, Gill TK, Taylor AW, Hill CL (2008). Predictors of podiatry utilisation in Australia: the North West Adelaide Health Study. J Foot Ankle Res.

[18] Wilson O, Kirwan J, Dures E, Quest E, Hewlett S (2017). The experience of foot problems and decisions to access foot care in patients with rheumatoid arthritis: a qualitative study. J Foot Ankle Res.

[19] cohealth https://www.cohealth.org.au/ Accessed 12 August 2021.

[20] Goeman D, Howard J, Ogrin R. Implementation and refinement of a community health nurse model of support for people experiencing homelessness in Australia: a collaborative approach. BMJ Open. 2019;9(11):e030982. 10.1136/bmjopen-2019-030982 PMC6887079 31748297

[21] Bolton Clarke. https://www.boltonclarke.com.au/additional-services/homeless-person-program Accessed 12 August 2021.

[22] Footscape http://www.footscape.com.au/. [Accessed 12 August 2021].

[23] Queens Nursing Institute. The foot health of people experiencing homelessness: Guidance for Community Nurses. 2020.

[24] IBM Corp. Released 2020. IBM SPSS statistics for windows VA, NY: IBM Corp.

[25] Mistry K, Ondhia C, Levell NJ (2020). A review of trench foot: a disease of the past in the present. Clin Exp Dermatol.

[26] Paudyal V, MacLure K, Buchanan C, Wilson L, Macleod J, Stewart D (2017). ‘When you are homeless, you are not thinking about your medication, but your food, shelter or heat for the night’: behavioural determinants of homeless patients' adherence to prescribed medicines. Public Health.

[27] City of Melbourne. homelessness-operating-protocol.pdf. [Accessed 12 August 2021].

[28] Adams L (2018). Melbourne, don't criminalise homelessness: working together to find alternatives to enforcement-based responses to homelessness. Parity..

[29] Army Public Health Center. Foot Marching, Load Carriage, and Injury Risk. 2016. 1010939.pdf (dtic.mil). [Accessed 12 August 21].

[30] Rayner J, Muldoon L, Bayoumi I, McMurchy D, Mulligan K, Tharao W (2018). Delivering primary health care as envisioned: a model of health and well-being guiding community-governed primary care organizations. J Integr Care (Brighton).

[31] Padgett D. Housing first: ending homelessness, transforming systems, and changing lives. Henwood BF, Tsemberis SJ, editors: New York, New York : Oxford University Press; 2016. 10.1176/appi.ps.671101 27903173

[32] Johnson G, Kuehnle D, Parkinson S, Sesa S, Tseng Y. Sustaining exits from long-term homelessness: a randomised controlled trial examining the 48 month social outcomes from the Journey to Social Inclusion pilot program. St. Kilda: Sacred Heart Mission; 2014.

[33] Wood L, Flatau P, Zaretzky K, Foster S, Vallesi S, Miscenko D. What are the health, social and economic benefits of providing public housing and support to formerly homeless people?, AHURI final report no.265, Australian Housing and Urban Research Institute, Melbourne. 2016. 10.18408/ahuri-8202801. http://www.ahuri.edu.au/research/final-reports/265. Accessed 26 Oct 21.

[34] AIHW (2015), Exploring transitions between homelessness and public housing, cat. No. HOU 277, Australian Institute of Health and Welfare, Canberra.

[35] Menant JC, Steele JR, Menz HB, Munro BJ, Lord SR (2008). Optimizing footwear for older people at risk of falls. J Rehabil Res Dev.

[36] Sherrington C, Menz HB (2003). An evaluation of footwear worn at the time of fall-related hip fracture. Age Ageing.

[37] Helquist L, Ogrin R, Rushford M-A, Mannix R, Lewis A (2020). Happy feet: a not-for-profit collaboration towards happier feet. Parity..

[38] Andersen RM (1995). Revisiting the behavioral model and access to medical care: does it matter?. J Health Soc Behav.

[39] Sheila D’Souza M, Mirza NA, Nairy KS (2021). Development of a foot care model to determine the risk of foot problems among homeless adults in Canada. Health Soc Care Community.

[40] Stafford A, Wood L (2017). Tackling health disparities for people who are homeless? Start with social determinants. Int J Environ Res Public Health.

